# Vegetarian Nutrition in Health Improvement

**DOI:** 10.3390/nu16234183

**Published:** 2024-12-03

**Authors:** Gianluca Rizzo, Luciana Baroni

**Affiliations:** 1 Independent Researcher, 98121 Messina, Italy; 2 Scientific Society for Vegetarian Nutrition—SSNV, 30171 Venice, Italy

There is a growing interest in vegetarian diets among the world population, and this has implications for public health. The current literature offers numerous sources for understanding the possible beneficial effects of plant-based foods on human health [1,2,3,4]. However, the wide variability of vegetarian diets, from the vegan diet that excludes any food of animal origin to the lacto-ovo-vegetarian one that includes dairy products and eggs, suggests the need to further expand knowledge on the subject. The Special Issue entitled “Vegetarian Nutrition in Health Improvement” aimed to collect experimental studies and literature reviews that could enrich the current state of the art on vegetarian nutrition and its effectiveness in improving health, to stimulate more effective policies for health and environmental sustainability, as well as prevention of chronic–degenerative diseases.

We know that the consumption of red meat, in particular processed meat, is associated with negative effects on health. For this reason, Niedermaier and colleagues conducted a 30-year simulation study on the impact of reducing or eliminating red meat and processed red meat on colorectal cancer (CRC) risk in Germany (Contribution 1). Currently, the mean consumption of red meat and processed meat among German men is 329 g and 427 g per week, respectively. Women have a lower consumption than men, with an age-standardized mean amount of 203 g and 224 g per week for red meat and processed meat, respectively. Reducing the mean consumption of both red meat and processed meat by 75 g or 150 g per week could reduce the burden of CRC by 1.1% and 6.5%, respectively. However, eliminating red meat and processed meat could reduce the incidence of CRC by 2.9% and 9.6%, respectively. This effect seems to be more pronounced among men compared to women.

The transition to a vegetarian diet also involves replacing animal-based foods with plant-based alternatives. For example, soymilk can be consumed as a substitute for cow’s milk. However, a lack of knowledge about the benefits of plant-based options, as well as concerns regarding taste, may pose obstacles to this transition. To identify potential predictors of soymilk consumption among the American population, Storz and colleagues conducted a cross-sectional study on participants from two different datasets of the National Health and Nutrition Examination Surveys (NHANES) (2015–2016 and 2017–2020, respectively) (Contribution 2). The total NHANES 2015–2016 sample for analysis comprised 5264 participants with a full dataset, of which 132 reported soymilk consumption. This may be extrapolated to represent 4,427,078 U.S. Americans. The NHANES 2017–2020 pre-pandemic cycle included 8511 participants with a full dataset, of which 187 reported soymilk consumption. This may be extrapolated to represent 3,460,784 U.S. Americans. In the multivariate logistic regression analysis, the authors found a direct association between soymilk consumption and moderate physical activity (OR: 2.36, 95% CI: 1.40–3.99, *p*: 0.003), as well as a high level of education (OR: 1.84, 95% CI: 1.01–3.33, *p*: 0.045). However, no association was observed between sex and soymilk consumption. Since individuals who consumed soy milk made up only 1.5 to 2% of the population sample, identifying strategies to promote soymilk consumption as a healthy choice for both personal health and the environment is essential.

Plant foods are not only a source of nutrients, but they also provide raw materials for the biotechnological extraction of health-beneficial molecules. This is the case of *Rhaponticum carthamoides* (Willd.) Iljin, a perennial plant belonging to the Asteraceae family, endemic to Eastern Europe, whose root extract (RCE) has been studied for its alleged benefits on lipid metabolism. In a comparative analysis conducted by Todorova and colleagues, RCE was tested in vitro on human preadipocytes (Contribution 3). The authors observed an anti-adipogenic and lipolytic effect by measuring the lipid accumulation and concentrations of free fatty acids and glycerol in the culture medium, respectively. The effect was due to both RCE and some secondary metabolites derived from it, whose presence was confirmed through the development and validation of a method based on high-performance thin-layer chromatography (HPTLC).

Although a plant-based diet has shown several health benefits, especially for chronic conditions, it must be well-planned. Among the at-risk nutrients often discussed, adequate protein intake is one of the most debated aspects. Plant proteins may have a reduced biological value compared to proteins of animal origin due to the presence of limiting amino acids and also due to the presence of fiber, protease inhibitors, and other plant matrix molecules, which reduce digestibility. To investigate this aspect, Bartholomae and Johnston conducted an intervention study on 17 minimally active vegan men who received a 5-day eucaloric diet containing 0.8 g/kg/d protein (Contribution 4). Protein sufficiency was assessed by nitrogen balance with 24 h urine collection on the fifth day of intervention. The authors observed a significant absolute and relative negative nitrogen balance (−1.38 g/d, 95% CI: −2.00 to −0.75, *p <* 0.001 and −18.60 mg/kg/d, 95% CI: −27.32 to −9.88, *p <* 0.001, respectively) that did not correlate with age, free-fat mass, or years being vegan. The results of this study confirm that a vegetarian diet, and in particular a vegan one, should ensure a higher protein intake than the U.S. recommended dietary allowance (RDA) for protein of 0.80 g/kg/d for adults, as also stated by experts and organizations in the field of vegetarian nutrition.

A vegetarian diet may be useful not only for the prevention and support of metabolic diseases but also for the management of disorders common in developed countries. Although the link between gastroesophageal reflux disease (GERD) and dietary choices remains unclear and understudied, some evidence suggests that nutrition may play a role in managing the disease both in the short and long term. Rizzo and colleagues explored this relationship through a cross-sectional study on 1077 adult participants of the INVITA study (Investigation on Italians’ Habits and Health) through a survey based on an online questionnaire (Contribution 5). In total, 37.3% of participants followed a vegan diet, with a prevalence of women, while the prevalence of GERD in the reference sample was 9%. As expected, the quality of life was statistically lower in individuals with GERD, based on the SF-36 questionnaire. Furthermore, the logistic multivariate analysis, adjusted for confounders such as BMI and smoking habits, showed that adopting a vegan diet compared to other animal-based dietary patterns significantly reduced the risk of GERD (OR: 0.47, 95% CI: 0.28–0.81, *p*: 0.006). This evidence suggests that a vegetarian diet can help improve the quality of life and manage disorders associated with GERD through simple lifestyle interventions.

Another aspect that affects the quality of life and long-term prognosis is frailty in senescence. This is relevant considering the increase in the aging population and the resulting health burden. Qi and colleagues conducted a prospective study including 2883 participants aged 65 years and above from the Chinese Longitudinal Healthy Longevity Survey (CLHLS) from 2008 to 2018 to explore the association between diet and frailty (through the frailty index) (Contribution 6). The authors assessed the participants’ eating habits through a food frequency questionnaire, defining the score for the overall plant-based diet index (PDI), healthful plant-based diet index (hPDI), and unhealthful plant-based diet index (uPDI). Using the Cox proportional hazard model corrected for covariates, a lower frailty risk was associated with adherence to a plant-based diet (HR: 0.86, 95% CI: 0.77–0.95, *p <* 0.01) and a healthful plant-based diet (HR: 0.83, 95% CI: 0.75–0.93, *p <* 0.001), when the highest tertile score was compared with the lowest. In contrast, adherence to an unhealthful plant-based diet was associated with a higher risk of frailty (HR: 1.21, 98% CI: 1.08–1.36, *p <* 0.01). While at baseline, only individuals without predisposition to frailty were included, at the end of the follow-up 1987 individuals suffered from pre-frailty or frailty, and the association between the frailty index and the plant-based diet indices was linear, with a more marked effect among men than women in the subgroup analysis (in women, statistical significance persisted only in the association with uPDI). This underlines how important it could be to plan a well-balanced vegetarian diet.

In a cross-sectional web-based survey with 2180 adult participants, Stenico and coworkers investigated the eating habits of an Italian sample living in Italy and abroad, following a vegan diet for at least one year (Contribution 7). Based on the results of structured and semi-structured questionnaires on food frequency, sociodemographics, and difficulties in daily management and relationships with health professionals, the participants were predominantly women and were motivated by ethical principles in their food choices. While ethical and environmental motivations were associated with the level of education, the duration of adherence to the diet was negatively associated with the environmental motivations and positively associated with the ethical ones. The length of time following the vegan diet was also positively associated with the consumption of plant-based cheeses and vegetable burgers and negatively associated with the consumption of cereals, legumes, and soy foods. Additionally, there was a strong awareness of the need for vitamin B12 supplements and a focus on following food guides for health. However, a skeptical approach from the medical community and health professionals emerged, raising concerns about the limited support for those who choose this dietary pattern.

Although the prevalence of plant-based foods in the diet has been reported to be associated with metabolic benefits, such as reduced cardiovascular risk, which food groups and macronutrients are specifically related to these benefits remains to be explored in depth. Jakše and colleagues conducted a secondary analysis from a Slovenian cross-sectional study (Whole-Food Plant-Based Diet Lifestyle Program) involving 151 individuals who had followed a well-structured plant-based diet for a long period (4.1 ± 2.5 years, on average) and maintained an active lifestyle (5541 ± 4677 total METs) (Contribution 8). From the multivariate analysis adjusted for confounders, the authors highlighted associations between foods such as fruits and whole grains and improved cholesterol levels, including total cholesterol (TC), high-density lipoprotein cholesterol (HDL-C), and low-density lipoprotein cholesterol (LDL-C). At the same time, an association between HDL-C levels and the consumption of legumes, nuts, and seeds was found. The study also pointed out associations between cholesterol levels and fiber, carbohydrates, and different types of fatty acids, even if the effects were weak (0.16 < r < 0.32). Moreover, some unexpected findings emerged, such as the negative association between HDL-C levels and higher consumption of legumes or nuts and seeds, as well as the lack of a significant association between food groups and blood pressure and the absence of a beneficial relationship between fiber intake and LDL-C levels. However, it must be noted that the study population had a homogeneous concentration of cardiovascular markers, which were within the normal range.

A narrative review and a systematic review were also included in this thematic collection.

Hernández-Lougedo and colleagues conducted a systematic review of the literature on the relationship between athletic performance and vegetarian nutrition (Contribution 9). After applying inclusion criteria, they selected six studies: three cross-sectional studies and three clinical trials. Two studies did not differentiate between types of vegetarian diets, one study focused on lacto-ovo-vegetarians, and the other three specifically involved participants following a vegan diet. Only one of the clinical trials was randomized and included a blinded omnivorous or vegetarian dietary intervention. Although overall differences in performance and health between diets were not evident, specific differences did emerge. The best performers in endurance activities (races under 21 km and half marathons) were individuals following a vegetarian diet, except for marathon or ultra-marathon events, where omnivores performed better. Omnivores showed better adaptation to environmental conditions, with significant differences observed between omnivorous and vegetarian women. Muscle mass in vegetarians was significantly lower than in omnivores. However, in cycle ergometer endurance tests, vegetarians showed a performance advantage over omnivores. No significant differences were observed regarding strength parameters. Given the still fragmentary nature of the data, further studies are needed to fully understand the influence of diet on physical performance, particularly in relation to specific parameters and sports disciplines.

A vegetarian diet has often been proposed as a beneficial dietary approach for chronic kidney disease (CKD), due to the greater tolerance of plant proteins compared to animal proteins and better compliance compared to the canonical low-protein diets commonly used in these cases. Narasaki and colleagues conducted a narrative review of the literature to investigate the available information on the potential influence of vegetarian nutrition on CKD (Contribution 10). A vegetarian diet has shown benefits for various conditions that exacerbate CKD, such as hypertension and diabetes. Additionally, it can be beneficial for reducing oxidative stress, inflammation, metabolic acidosis, and the accumulation of uremic toxins. Many of the benefits of plant-based foods come from the presence of phytochemicals and fiber. The adoption of a vegetarian diet in CKD patients has been debated due to the high phosphorus and potassium content in plant foods, which could worsen the renal burden associated with mineral metabolism disorders. However, the literature data suggest that phosphorus in plant foods is much less absorbable and bioavailable than that found in animal products. Phosphorus in vegetables is complexed with macromolecules that reduce the risk of hyperphosphatemia, as demonstrated in comparative studies with similar intake levels between plant and animal foods. Similarly, the risk of hyperkalemia does not seem relevant enough to justify the avoidance of plant foods, except for some processed items such as fruit juices, dried fruits, and sauces. The relationship between diet and end-stage kidney disease remains to be clarified.

Overall, plant-based diets appear to have positive implications for health, if appropriate adjustments are made compared with guidelines for the general population. Health professionals can play a crucial role in managing vegetarian diets, avoiding misinformation, and promoting sustainability for individuals, society, and the environment. Consumers’ knowledge about plant-based foods needs to be enhanced.
